# Differential Gene Expression Changes in Children with Severe Dengue Virus Infections

**DOI:** 10.1371/journal.pntd.0000215

**Published:** 2008-04-09

**Authors:** Martijn D. de Kruif, Tatty E. Setiati, Albertus T. A. Mairuhu, Penelopie Koraka, Hella A. Aberson, C. Arnold Spek, Albert D. M. E. Osterhaus, Pieter H. Reitsma, Dees P. M. Brandjes, Augustinus Soemantri, Eric C. M. van Gorp

**Affiliations:** 1 Department of Internal Medicine, Slotervaart Hospital, Amsterdam, The Netherlands; 2 Center for Experimental and Molecular Medicine, Academic Medical Center, University of Amsterdam, The Netherlands; 3 Department of Pediatrics, Dr. Kariadi Hospital, Semarang, Indonesia; 4 Institute of Virology, Erasmus Medical Centre, Rotterdam, The Netherlands; Instituto de Medicina Tropical Pedro Kouri, Cuba

## Abstract

**Background:**

The host response to dengue virus infection is characterized by the production of numerous cytokines, but the overall picture appears to be complex. It has been suggested that a balance may be involved between protective and pathologic immune responses. This study aimed to define differential immune responses in association with clinical outcomes by gene expression profiling of a selected panel of inflammatory genes in whole blood samples from children with severe dengue infections.

**Methodology/Principal Findings:**

Whole blood mRNA from 56 Indonesian children with severe dengue virus infections was analyzed during early admission and at day −1, 0, 1, and 5–8 after defervescence. Levels were related to baseline levels collected at a 1-month follow-up visit. Processing of mRNA was performed in a single reaction by multiplex ligation-dependent probe amplification, measuring mRNA levels from genes encoding 36 inflammatory proteins and 14 Toll-like receptor (TLR)-associated molecules. The inflammatory gene profiles showed up-regulation during infection of eight genes, including *IFNG* and *IL12A*, which indicated an antiviral response. On the contrary, genes associated with the nuclear factor (NF)-κB pathway were down-regulated, including *NFKB1, NFKB2, TNFR1, IL1B, IL8,* and *TNFA*. Many of these NF-κB pathway–related genes, but not *IFNG* or *IL12A,* correlated with adverse clinical events such as development of pleural effusion and hemorrhagic manifestations. The TLR profile showed that TLRs were differentially activated during severe dengue infections: increased expression of *TLR7* and *TLR4R3* was found together with a decreased expression of *TLR1*, *TLR2*, *TLR4R4,* and TLR4 co-factor *CD14*.

**Conclusions/Significance:**

These data show that different immunological pathways are differently expressed and associated with different clinical outcomes in children with severe dengue infections.

## Author Summary

Dengue virus infection is an impressively emerging disease that can be fatal in severe cases. It is not precisely clear why some patients progress to severe disease whereas most patients only suffer from a mild infection. In severe disease, a “cytokine storm” is induced, which indicates the release of a great number of inflammatory mediators (“cytokines”). Evidence suggested that a balance could be involved between protective and pathologic cytokine release patterns. We studied this concept in a cohort of Indonesian children with severe dengue disease using a gene expression profiling method.

The study showed that the immune response to severe dengue infection was characterized by up-regulation of interferon pathway–associated cytokines and down-regulation of nuclear factor (NF)-kappaB pathway–associated cytokines. Many of the NF-kappaB pathway–related genes, but not the interferon pathway–associated genes, correlated with adverse clinical events such as development of pleural effusion and hemorrhagic manifestations. In addition, the study showed that the expression of specific pathogen recognition receptors called Toll-like receptors was differentially regulated in patients with dengue. Together, the data show that different immunological pathways may indeed be differently expressed and associated with different clinical outcomes in children with severe dengue infections.

## Introduction

Dengue disease is emerging in the developing world at an alarming rate [1]. Moreover, the disease is potentially lethal, but only supportive measures are available. A major obstacle in the development of novel therapeutic strategies is that the pathophysiology of dengue disease is poorly understood [2],[3]. Clinically, dengue disease is a mosquito-borne disease especially affecting children in endemic, mostly tropical regions and is caused by infection with dengue virus, a member of the *Flaviviridae* family [4]. Though most patients only suffer from a mild febrile illness called dengue fever (DF), a relatively small group of patients experiences more severe forms of disease which are characterized by increased vascular permeability leading to bleeding manifestations and shock, such as dengue hemorrhagic fever (DHF) and dengue shock syndrome (DSS) [5].

The mechanism leading to severe, critical disease is insufficiently understood. Antibody-dependent enhancement of viral replication is generally thought to play a role since most cases of DHF/DSS occur after secondary infection by a dengue virus serotype different from the first infection [2], [6]–[8]. Severe disease is characterized by high viremia titers, coagulation abnormalities and activation of the immune system [5],[9]. The host defense strongly depends on interferon (IFN)-γ production, but other cytokines are also involved, as well as antibody production and T cell responses [10],[11]. Typically, cytokine levels are highest among the day of defervescence, which is defined as the first day after the start of fever that the body temperature returns to normal. At the same day viremia titers drop to low or undetectable levels, whereas the risk of shock development is increased [12]. The relationship between cytokine production and severe disease is not fully understood, but it is increasingly recognized that a balance is involved between protective and pathologic immune responses [2]. In general, cytokine production depends on the recognition of pathogens via Toll-like receptors (TLRs). In viral disease, TLR3, TLR7, TLR8 and TLR9 are commonly involved, but specific knowledge about TLRs in dengue infections is limited [13]–[15].

RNA-based gene expression profiling methods offer the opportunity to examine complex biological processes in a large context. We previously developed and validated an RT-PCR based assay for the measurement of multiple mRNA levels in a single reaction, multiplex ligation-dependent probe amplification (MLPA) [16]–[19]. The MLPA panel includes 36 target genes of various mediators of inflammation, such as cytokines, chemokines and intracellular signalling molecules and in addition a second panel has been developed to measure 14 TLR associated genes [20].

In order to investigate patterns of innate immune responses in severe dengue infections in children at the level of gene transcription, blood samples were collected from a group of children with severe dengue disease in Indonesia. The samples were analyzed using MLPA technology and associated with clinical outcomes.

## Materials and Methods

### Study design

The study was designed as a prospective, observational cohort study and was conducted in the Dr. Kariadi University Hospital in Semarang, Indonesia, simultaneously with an other study investigating the pathophysiology of hemorrhagic tendencies in dengue virus infections [21]; from this cohort, only patients were included for whom RNA was successfully isolated, which was available for all patients consecutively admitted from February 2002 until March 2003. The ethics committee/ institutional review board of the dr Kariadi University Hospital in Semarang, Indonesia, approved all legal, ethical, clinical and laboratory aspects of the study including the informed consent procedure, which was obtained, after informing the patients and their parents and/or guardians in local language, as a written form from children's parents or guardians before inclusion. Children, aged 3 to 14 years, admitted to the paediatric intensive care unit or a ‘high nursery care unit’ with a clinical diagnosis of suspected DHF or DSS were included. Demographic and clinical data were collected using a standardized data collection form. A Tourniquet test was performed to detect a bleeding tendency together with chest- and abdominal X-rays and ultrasonography to detect ascites and/or pleural effusion. Children were classified as having DF, DHF or DSS according to WHO criteria [1]. In brief, the clinical diagnosis of DHF included the presence of fever, a hemorrhagic tendency, thrombocytopenia (<100×10^3^/mm^3^), evidence of pleural effusion and/or a significant (>20%) rise, or drop after volume replacement therapy, of hematocrit values. DSS was defined as DHF with evidence of circulatory failure. All other cases not meeting these criteria were diagnosed DF. Citrated blood samples were obtained on the day of admission, days 1, 2 and 7 after admission and at a 1-month follow-up visit. Because in dengue disease each patient experiences a different clinical course and is hospitalized at different time points in that course, these blood samples were referred to a common clinical time point, which is the day of defervescence according to international agreement. In addition, samples obtained at admission from patients presenting >1 day before defervescence were clustered into a time point designated ‘Early’, which indicates early immune activation before defervescence.

### Diagnostic procedures

Paired blood samples were tested for serologic evidence of acute dengue infection. Dengue virus specific IgG and IgM antibodies were measured by ELISA (Focus Technologies, Cypress, Ca, USA). The sensitivity and specificity of these tests were evaluated previously [22]. Cases were considered serologically-confirmed if the IgM ELISA was positive during the acute phase of disease (optical density of the sample higher than the optical density of the cut-off serum provided by the manufacturer) and/or if a four-fold increase in IgG titre was demonstrated in paired acute and convalescent sera. For some patients, a definitive serodiagnosis was not possible because no convalescent sample was obtained. Dengue virus antigen and/or viral RNA was detected in these cases using a dot blot immunoassay and/or a dengue serotype specific reverse transcription PCR, respectively [23]. Patients with definitive serodiagnosis and/or positive dot blot and/or positive PCR were considered to have confirmed dengue virus infection.

### mRNA analysis

Blood samples were centrifuged within 1–2 hours after retrieval at 15°C for 20 minutes at 1600×*g*. Subsequently, plasma was separated and red blood cells were lysed using Red Blood Cell lysis Buffer (Roche, Mannheim, Germany) according to the manufacturer's protocol. Total white blood cells were stored at −80°C in mRNA lysis/-binding buffer (Roche) and transported to the Netherlands on dry ice where total mRNA was isolated using High Pure RNA Isolation Kit (Roche). The mRNA of multiple inflammatory molecules was analyzed by MLPA as described previously [16]. In addition a second panel was added measuring the involvement of multiple TLR related genes [20]. MLPA is insensitive to the total amount of mRNA that is included in the reaction; therefore, the profile is independent of the total white blood cell (WBC) count. All samples were tested with the same batches of reagents, and a negative and endotoxin-stimulated control sample were included on each plate. The final polymerase chain reaction (PCR) fragments amplified with carboxyfluorescein-labeled primers were separated by capillary electrophoresis on a 16-capillary ABI-Prism 3100 Genetic Analyzer (Applied Biosystems, Nieuwerkerk aan de IJssel, The Netherlands). Peak area and height were processed using GeneScan analysis software (Applied Biosystems). The levels of mRNA for each gene were expressed as a normalized ratio of the peak area divided by the peak area of a control gene, beta 2 microglobulin (*B2M*), resulting in the relative abundance of mRNAs of the genes of interest.

### Statistical analysis

All calculations were carried out using SPSS version 13.0 (SPSS Inc., Chicago, IL, USA). The relative mRNA levels of subjects from time-point ‘Early’ and day −1, 0, 1 and 5–8 post defervescence were compared to baseline levels collected at a 1-month follow up visit using Wilcoxon signed ranks test. mRNA's with >80% of values below the detection limit were excluded from analysis and considered not detectable. *P*-values for each time point were corrected for multiple comparisons according to the method of Benjamini and Hochman, with q = 0.1 [24]. Associations with clinical variables were calculated by univariate logistic regression analysis and odds ratios±SE are presented. A P<0.05 was considered statistically significant.

## Results

### Patient characteristics

Samples for mRNA analysis were collected from 56 children with confirmed dengue virus infections. All children presented with clinically severe disease; the patients were classified according to WHO criteria [1] as having DF (n = 7; 13%), DHF (n = 29; 51%) or DSS (n = 20; 36%). Twenty patients presented more than 1 day before defervescence (median 3 days before defervescence; interquartile range 2–3). These patients had experienced symptoms since 4 days before defervescence (median; interquartile range 3–5). Further patient characteristics at admission are given in Table 1. During stay at the hospital, 10 patients (18%) developed one or more complications related to their dengue infections. Profound shock was noted in 7 children (13%) and recurrent shock in 5 children (9%). Other complications included disseminated intravascular coagulation (n = 3; 5%), encephalopathy (n = 3; 5%) or pulmonary edema (n = 1; 2%). Eventually, 4 patients (7%) died despite treatment. These patients had all been classified as having DSS; two of them died from profound shock; one patient also had recurrent shock and encephalopathy and one patient suffered in addition from disseminated intravascular coagulation. The surviving patients all made a complete recovery within a maximum of 12 days of stay in the hospital.

**Table 1:** Clinical characteristics of 56 children from Indonesia admitted to the hospital with severe dengue virus infections.

	All (n = 56)	DF/DHFa) (n = 36)	DSSa) (n = 20)
Age, median (IQR), years	6 (5–9)	7 (5–10)	6 (5–7)
Male sex, n (%)	22 (39)	14 (39)	8 (40)
Primary infectionb), n (%)	4 (7)	4 (11)	0 (0)
Duration of symptoms before admission, median (IQR), days	4 (3–4)	3 (3–4)	4 (3–5)
Positive Tourniquet test, n (%)	33 (59)	27 (75)	6 (30)
Petechiae, ecchymoses or purpura, n (%)	28 (50)	18 (50)	10 (50)
Mild spontaneous bleeding, n (%)	15 (27)	9 (25)	6 (30)
Severe bleeding (hematemesis and/or melena), n (%)	5 (9)	2 (6)	3 (15)
Thrombocytopenia (platelet count <100 10^3^/mm^3^), n (%)	52 (93)	32 (89)	20 (100)
Hematocrit >20% above normal for age, n (%)	11 (20)	7 (19)	4 (20)
Hematocrit >20% drop after volume replacement, n (%)	16 (29)	10 (28)	6 (30)
Pleural effusion, n (%)	44 (79)	26 (72)	18 (90)
Ascites, n (%)	19 (34)	11 (31)	8 (40)
Hypoproteinaemia (<6.0 g/L), n (%)	25 (45)	16 (44)	9 (45)
Hypotension for age, n (%)	15 (27)	6 (17)	9 (45)
Pulse pressure ≤20 mmHg, n (%)	24 (43)	10 (28)	14 (70)
Heart frequency, median (IQR), min^−1^	120 (112–132)	120 (110–131)	122 (120–132)
Duration of hospital stay, median (IQR), days	5 (3–6)	4 (3–5)	6 (3–7)
Mortality, n (%)	4 (7)	0 (0)	4 (20)

a) dengue fever (DF), dengue hemorrhagic fever (DHF) and dengue shock syndrome (DSS) according to WHO criteria

b) primary or secondary infection

### Inflammatory gene expression profiling

**Figure 1:**
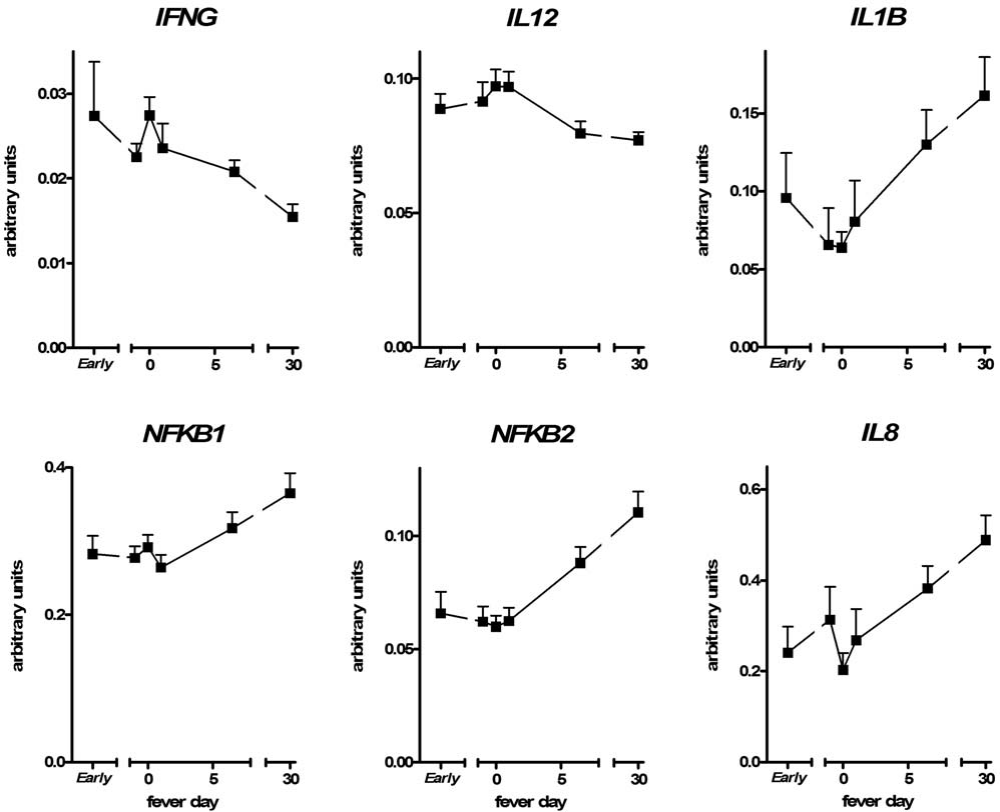
Differential gene expression patterns in dengue disease. mRNA levels (arbitrary units relative to β-2-microglobulin expression; presented as mean±SEM) from Indonesian children (n = 56) with severe dengue infections from day ‘Early’ (including admission samples of patients presenting >1 day before defervescence; n = 20), −1, 0, 1 and 5–8 from defervescence. Whereas *IFNG* and *IL12A* were up-regulated, a cluster of genes related to the NF-κB pathway was down-regulated, including *NFΚB1, NFΚB2, IL1B* and *IL8*.

Gene expression profiles of 36 inflammatory genes during hospital stay of each fever day were compared to baseline values at a 1-month follow up visit (Table 2). The profile showed up-regulation of 8 genes, down-regulation of 7 genes and no effect on 13 genes, whereas 8 genes were not detectable. Most gene expression profile changes occurred on the day of defervescence (day 0). The expression patterns revealed up-regulation of the Th1 type cytokines interferon-γ (*IFNG*) and interleukin-12a (*IL12A*) on consecutive days, indicating an antiviral response. Another gene strongly induced by dengue infection was *MIF*, a marker of macrophage activation. No other cytokine genes from the MLPA panel were up-regulated, but some other genes were involved, including cell cycle control genes such as cyclin-dependent kinase inhibitor 1A (*CDKN1A*) at consecutive days and polyadenylate-specific ribonuclease *(PARN)* and protein-tyrosine phophatase nonreceptor-type 1 (*PTPN1)* at day 0 only. Furthermore, glutathione S-transferase (*GSTP1*), involved in detoxification metabolism, was significantly up-regulated at day −1 and day 1 and *MD2*, a co-factor of TLR4, was activated early before defervescence. Of note, in contrast to the enhanced levels of antiviral cytokine mRNAs, down-regulation of genes was observed at consecutive days for a whole cluster of genes associated with the nuclear factor (NF)-κB pathway (Figure 1). Transcription levels of NF-κB subunit 2 (*NFΚB2*) and tumor necrosis factor receptor 1 (*TNFR1*) were decreased as well as levels of the cytokine genes *IL1B* at day −1 and *IL8, TNFA* and NF-κB subunit 1 (*NFΚB1*) at day 0. Furthermore, transcription was inhibited during the early phase of *PTPN4A2*.

**Table 2:** Inflammatory gene expression profiling of 56 children from Indonesia with severe dengue virus infections: *P*-values indicating up- or down-regulation relative to baseline.

Gene transcription response	Gene symbol	Descriptive name	*P*-valueb)				
			Daya) *E*	Daya) -1	Daya) 0	Daya) 1	Daya) 5–8
Up	*CDKN1A*	Cyclin-dependent kinase inhibitor, 1A	0.008	0.033	0.002	0.013	0.017
	*GSTP1*	Glutathione S-transferase	NS	0.016	NS	0.008	NS
	*IFNG*	Interferon, gamma	NS	0.009	0.002	0.043	0.015
	*IL12A*	Interleukin 12, subunit p35	NS	NS	0.002	0.005	NS
	*MD2*	MD2 protein	0.011	NS	NS	NS	NS
	*MIF*	Macrophage migration inhibitory factor	0.041	0.000	0.002	0.000	0.018
	*PARN*	Polyadenylate-specific ribonuclease	NS	NS	0.016	NS	NS
	*PTPN1*	Protein-tyrosine phophatase, nonreceptor-type 1	NS	NS	0.021	NS	NS
Down	*IL1B*	Interleukin 1, beta	0.029	0.016	NS	NS	NS
	*IL8*	Interleukin 8	0.010	NS	0.004	NS	NS
	*NFKB1*	Nuclear factor kappa-B, subunit 1	NS	NS	0.043	NS	NS
	*NFKB2*	Nuclear factor kappa-B, subunit 2	0.002	0.002	0.005	0.001	NS
	*PTP4A2*	Protein-tyrosine phophatase, type 4a, 2	0.007	NS	NS	NS	NS
	*TNFA*	Tumor necrosis factor, alpha	NS	NS	0.016	NS	NS
	*TNFR1*	Tumor necrosis factor receptor superfamily 1a	0.002	0.001	0.002	0.011	NS
Non significant	*BMI1*	BMI-1 oncogene homolog	NS	NS	NS	NS	NS
	*IL1RA*	Interleukin 1 receptor antagonist	NS	NS	NS	NS	NS
	*IL4*	Interleukin 4	NS	NS	NS	NS	NS
	*IL15R1*	Interleukin 15, transcript variants 1 and 3	NS	NS	NS	NS	NS
	*LTA*	Lymphotoxin alfa	NS	NS	NS	NS	NS
	*MCP1*	Monocyte chemotactic protein 1	NS	NS	NS	NS	NS
	*MIP1A*	Macrophage inflammatory protein 1, alpha	NS	NS	NS	NS	NS
	*MIP1B*	Macrophage inflammatory protein 1, beta	NS	NS	NS	NS	NS
	*MYC*	Oncogene MYC	NS	NS	NS	NS	NS
	*NFKB1A*	Nuclear factor kappa-B inhibitor alpha	NS	NS	NS	NS	NS
	*PDE4B*	Phosphodiesterase 4B, cAMP specific	NS	NS	NS	NS	NS
	*SERPB9*	Serine proteinase inhibitor, clade B, member 9	NS	NS	NS	NS	NS
	*THBS1*	Thrombospondin 1	NS	NS	NS	NS	NS
Not detectable	*IL1A*	Interleukin 1, alpha	ND	ND	ND	ND	ND
	*IL2*	Interleukin 2	ND	ND	ND	ND	ND
	*IL6*	Interleukin 6	ND	ND	ND	ND	ND
	*IL10*	Interleukin 10	ND	ND	ND	ND	ND
	*IL18*	Interleukin 18	ND	ND	ND	ND	ND
	*MCP2*	Monocyte chemotactic protein 2	ND	ND	ND	ND	ND
	*PDGFB*	Platelet-derived growth factor beta	ND	ND	ND	ND	ND
	*TF*	Tissue factor	ND	ND	ND	ND	ND

a) day *E*, −1, 0, 1 and 5–8 post defervescence; defined as the first day after start of fever that the body temperature returns to normal. *E* = Early; defined as clustering of admission samples of patients presenting >1 day before defervescence (n = 20).

b) *P*-values indicate up- or down-regulation of gene transcription during infection, relative to baseline levels collected from a 1-month follow-up visit. A *P*-value<0.05 was considered statistically significant. NS = Not Significant. ND = Not Detectable.

### TLR gene expression profiling

The gene expression profiles of 14 TLR associated genes during admission showed up-regulation of 2 genes, down-regulation of 4 genes and no effect on 6 genes, whereas 2 genes were not detectable (Table 3). Most gene expression profile changes occurred at day −1 and day 0. Up-regulation of TLR gene transcription was observed for *TLR7* and TLR4 transcript variant 3 (*TLR4R3*) during the early phase and at day −1; in addition, *TLR7* was up-regulated at day 0 and *TLR4R3* at day 1. Decreased expression of TLRs was noted for *TLR1*, *TLR4R4* and TLR4 co-factor *CD14* at day −1 and day 0, whereas decreased expression of *TLR4R4* was also noted during early activation and for *TLR2* at all time-points. No detectable levels were measured of the viral recognition associated TLR genes *TLR3* and *TLR9*.

**Table 3:** TLR gene expression profiling of 56 children from Indonesia with severe dengue virus infections: *P*-values indicating up- or down-regulation relative to baseline.

Gene transcription response	Gene symbol	Descriptive name	*P*-valueb)				
			Daya) *E*	Daya) −1	Daya) 0	Daya) 1	Daya) 5–8
Up	*TLR4R3*	Toll-like receptor 4, transcript variant 3	0.003	0.000	NS	0.030	NS
	*TLR7*	Toll-like receptor 7	0.001	0.017	0.013	NS	NS
Down	*CD14*	Monocyte differentiation antigen CD14	NS	0.002	0.044	NS	NS
	*TLR1*	Toll-like receptor 1	NS	0.035	0.002	NS	NS
	*TLR2*	Toll-like receptor 2	0.026	0.009	0.005	0.048	0.043
	*TLR4R4*	Toll-like receptor 4, transcript variant 4	0.005	0.030	0.010	NS	NS
Non significant	*TLR4R1*	Toll-like receptor 4, transcript variant 1	NS	NS	NS	NS	NS
	*TLR4R2*	Toll-like receptor 4, transcript variant 2	NS	NS	NS	NS	NS
	*TLR5*	Toll-like receptor 5	NS	NS	NS	NS	NS
	*TLR8R1*	Toll-like receptor 8, transcript variant 1	NS	NS	NS	NS	NS
	*TLR8R2*	Toll-like receptor 8, transcript variant 2	NS	NS	NS	NS	NS
	*TLR10*	Toll-like receptor 10	NS	NS	NS	NS	NS
Not detectable	*TLR3*	Toll-like receptor 3	ND	ND	ND	ND	ND
	*TLR9*	Toll-like receptor 9	ND	ND	ND	ND	ND

a) day *E*, −1, 0, 1 and 5–8 post defervescence; defined as the first day after start of fever that the body temperature returns to normal. *E* = Early; defined as clustering of admission samples of patients presenting >1 day before defervescence (n = 20).

b) *P*-values indicate up- or down-regulation of gene transcription during infection, relative to baseline levels collected from a 1-month follow-up visit. A *P*-value<0.05 was considered statistically significant. NS = Not Significant. ND = Not Detectable.

### Clinical associations

Clinical associations of gene expression levels with parameters of disease severity were calculated at day −1, 0 and 1 from defervescence. Odds ratios and p-values are presented in Table 4. The development of DSS correlated significantly with down-regulation of *IL4R2*. Pleural effusion as diagnosed by either chest X-ray or ultrasonography was associated with up-regulation of members of the NF-κB pathway such as *TNFA* and *TNFR1*, but also with *IL1RA*, *MIP1A* and *TLR1* and was inversely related to *MIF* and *TLR7*. Hemorrhagic manifestations including mild and/or severe bleeding were associated with the NF-κB pathway members *NFKB1* and *TNFR1.* The occurrence of severe complications such as profound or recurrent shock, disseminated intravascular coagulation, encephalopathy and/or pulmonary edema was not significantly associated with genes from the panel but *CD14*, *IL1B, IL1RA*, *NFKB1*, *PDE4B*, *TLR1* and *TLR10* showed a trend towards significance, particularly during defervescence.

**Table 4:** Association of inflammatory gene expression levels with disease severity parameters in 56 children from Indonesia with severe dengue virus infections during defervescence.

Parameter		Daya) −1			Daya) 0			Daya) 1		
	Marker	ORb)	SEb)	*P* b)	ORb)	SEb)	*P* b)	ORb)	SEb)	*P* b)
**DSS**	***IL4R2***	−7.22	5.26	0.001*	NS	NS	NS	NS	NS	NS
	***PTPN1***	NS	NS	NS	NS	NS	NS	37.7	20.6	0.067
**Complications**	***CD14***	NS	NS	NS	0.624	0.375	0.097	NS	NS	NS
	***IL1B***	NS	NS	NS	23.0	13.9	0.099	NS	NS	NS
	***IL1RA***	NS	NS	NS	11.8	7.00	0.091	NS	NS	NS
	***NFKB1***	NS	NS	NS	35.4	21.4	0.098	NS	NS	NS
	***PDE4B***	NS	NS	NS	NS	NS	NS	6.19	3.32	0.063
	***TLR1***	NS	NS	NS	2.16	1.12	0.053	NS	NS	NS
	***TLR10***	NS	NS	NS	16.5	9.78	0.092	NS	NS	NS
**Pleural effusion**	***CD14***	NS	NS	NS	0.878	0.504	0.082	0.848	0.476	0.075
	***IL1RA***	NS	NS	NS	21.6	9.89	0.029*	9.75	5.82	0.094
	***IL4R2***	NS	NS	NS	38.6	22.7	0.09	NS	NS	NS
	***IL8***	NS	NS	NS	11.4	60.1	0.061	NS	NS	NS
	***MIF***	NS	NS	NS	−10.9	5.39	0.043*	−13.0	5.47	0.017*
	***MIP1A***	NS	NS	NS	NS	NS	NS	217.1	104	0.037*
	***NFKB2***	NS	NS	NS	64.4	36.2	0.075	NS	NS	NS
	***TLR1***	2.13	1.07	0.046*	NS	NS	NS	NS	NS	NS
	***TLR4R4***	6.59	3.29	0.045*	13.7	6.95	0.049*	10.6	6.04	0.077
	***TLR7***	−4.05	1.98	0.041*	−5.24	2.55	0.038*	NS	NS	NS
	***TNFA***	NS	NS	NS	118.3	59.6	0.047*	NS	NS	NS
	***TNFR1***	13.4	7.53	0.075	31.4	15.9	0.048*	12.5	6.81	0.067
**Hemorrhagic manifestations**	***IL4R2***	NS	NS	NS	NS	NS	NS	7.18	4.31	0.095
	***NFKB1***	NS	NS	NS	NS	NS	NS	14.0	6.42	0.029*
	***PTP4A2***	NS	NS	NS	NS	NS	NS	5.03	2.77	0.07
	***TLR5***	11.0	6.33	0.083	NS	NS	NS	NS	NS	NS
	***TNFR1***	NS	NS	NS	NS	NS	NS	14.0	6.86	0.041*

a) Day −1, 0 and 1 post defervescence; defined as the first day after start of fever that the body temperature returns to normal.

b) OR±SE = odds ratio±standard error. A *P*-value <0.05 was considered statistically significant (^*^). P-values showing a trend to significance (<.10) are also shown. NS = Not Significant.

## Discussion

The pathogenesis of severe critical disease following dengue virus infection involves the activation of multiple inflammatory pathways. This is the first study to report high throughput gene expression profile changes of inflammatory genes in children with severe dengue virus infections. The study revealed two points of interest. First, the profile showed characteristics of a general antiviral response with up-regulation of *IFNG*, which is an established major antiviral cytokine in dengue disease, together with up-regulation of *IL12A*, a potent stimulator of IFN-γ production [11],[25]. In contrast, no other cytokine genes from the panel were up-regulated, but a cluster of genes related to the NF-κB pathway was down-regulated, including *NFΚB1, NFΚB2, TNFR1, IL1B, IL8* and *TNFA*. In addition, the NF-κB pathway linked expression patterns of *NFΚB1, TNFR1* and *TNFA* correlated with adverse clinical events, such as the development of pleural effusion and hemorrhagic manifestations. A second finding of interest in this study is that this is the first report of *in vivo* differential activation of multiple TLRs in dengue disease, which may implicate a role for these receptors in dengue infections.

The intracellular NF-κB pathway is a major route for inflammatory stimuli to the release of most pro-inflammatory mediators, including TNF-α, IL-1ß and IL-8 [26]. Most of these mediators were reported to be elevated in plasma from patients with severe dengue disease, especially around the day of defervescence [27],[28]. In contrast, studies showed poor NF-κB related cytokine production after stimulation of peripheral blood mononuclear cells which were isolated from patients with dengue [29]–[31]. Also in the current study most *in vivo* NF-κB pathway associated gene transcription levels were down-regulated. These results may be limited to the site of production, i.e. located to circulating leucocytes, because *in vitro* data showed that dengue virus was able to stimulate the NF-κB pathway in other, non leukocyte cells, such as human hepatoma cells and endothelial cells [32],[33]. Yet, no studies have directly compared different cell types or investigated the mechanism or function of NF-κB down-regulation by dengue. Down-regulation of inflammatory genes during the day of defervescence may also be the result of time effects such as depletion- or negative feed-back mechanisms following previous activation of these genes. Time effects play an important role in dengue disease; the immune response can be roughly divided into early inflammatory changes, changes during defervescence and late changes after defervescence [12],[34]. An exploration of ‘early’ time effects was carried out in our study by clustering all early admission samples from patients entering the hospital more than one day before defervescence. However, because no major differences were noted in comparison to changes observed during defervescence, it shows no evidence that early time effects affecting the investigated genes interfered with later changes during defervescence.

The production of cytokines such as INF-γ indicates activation of an appropriate, protective immune response to dengue virus infection. However, overwhelming production of cytokines and/or cross-activation of pathological immune responses has been postulated as a mechanism to endothelial damage and subsequent vascular leakage, shock and death. Previous studies showed direct associations between mortality and increased protein plasma levels of IL-6, IL-8, IL-10, IL-1RA and MIF [31],[35],[36]. In addition, increased plasma levels of TNF-α, TNFR1, TNFR2, IL2R, IL-8, IL-13, IL-18, soluble CD8 and TGFß1 protein plasma levels were associated with development of DSS, whereas levels of TNF-α also correlated with hemorrhagic manifestations and levels of TNFR1 were associated with plasma leakage [27], [29], [35], [37]–[40]. Within this context, it is important to define which immune responses are protective and which responses play a mere pathological role. Clustering of the confusingly large amount of mediators which appear to be involved in dengue into collective pathways can help to retrieve a clearer picture, if possible. A previous microarray based study revealed this way that activation of interferon-I dependent pathways was associated with beneficial outcomes in adult patients with dengue [41]. The current study did not investigate interferon-I dependent pathways, but included the genes *IFNG* and *IL12A* and found no evidence of an association of these genes with adverse outcomes. In contrast, the current study found associations of various other inflammatory genes with adverse outcomes and it showed that at least for some part these associations could be clustered to members of the NF-κB pathway: *TNFA* and *TNFR1* were highly associated with pleural effusion (odds ratios 118.3±59.6 and 31.4±15.9, respectively) and *NFKB1* and *TNFR1* correlated with hemorrhagic manifestations (odds ratios 14.0±6.4 and 14.0±6.9, respectively). In addition, a trend towards significance was found for association of the NF-κB pathway related genes *IL1B* and *NFKB1* with the occurrence of severe complications and for *IL8* and *NFKB2* with pleural effusion. Of note, TNF-α is one of the major NF-κB pathway effector molecules and others showed that in two independent mouse models of dengue infection, antibodies to TNF-α significantly decreased dengue disease severity and mortality *in vivo* [42],[43]. Also, it was demonstrated that addition of serum from patients with acute dengue infection to endothelial cells *in vitro* induced increased expression of the endothelial cell activation marker ICAM-1 and that this effect could be blocked by antibodies to TNF-α [44]. Based upon these data all together, activation of TNF-α in particular, but possibly of the NF-κB pathway as a whole, may be involved in the mechanism of vascular leakage and transition into severe disease in patients with dengue.

Pathogen recognition is an important step in the activation of inflammatory pathways and TLRs play a major role in this process [26]. Each TLR is associated with specific ligands and response patterns, though interactions occur and in fact each pathogen is recognized by not only one TLR but by a set of TLRs, usually together with also other pattern-recognition receptors [45]. In the current study, the *in vivo* role of ten TLRs during dengue infection was explored and differential gene expression of TLRs was found: increased expression of *TLR7* and *TLR4R3* together with a decreased expression of *TLR1*, *TLR2*, *TLR4R4* and TLR4 co-factor *CD14*. These results show that the transcription profile of the total white blood cells is perturbed in children with severe dengue disease; the lack of clustered data hampers further interpretation of these results. The *in vivo* role of TLR7 in particular though, which was up-regulated in our panel and associated with a lack of pleural effusion in patients during fever day -1 and day 0 (odds ratios 4.05±1.98 and 5.24±2.55, respectively) may be of interest to be further explored, also because previous investigators identified TLR7 as an endosomal pattern-recognition receptor for single-stranded RNA viruses including influenza virus and vesicular stomatitis virus [14],[46] and a recent study showed that TLR7 could be triggered by dengue virus *in vitro* [47].

Gene profiling methods offer opportunities to examine biological processes in great detail. Yet, due to the amount of data and because mRNA levels may be subject to posttranslational processing there is risk of over interpretation of results. Moreover, because the current study is an observational study, it has difficulty to distinguish between primary and secondary effects [48]. Still, the current MLPA panel was validated before and proved useful for the study of molecular mechanisms during various inflammatory conditions [16]–[20]. The statistical error in the present study was reduced as much as possible by applying Benjamini-Hochman statistical correction for multiple testing [24]. Furthermore, the error was reduced by testing at several days independently from each other: still, most gene expression changes occurred at several days consecutively. In addition, most changes were noted at the day of defervescence with a more or less gradual decline of changes during recovery; this pattern closely resembles data from others [27],[34]. As such, the data suggest that the observed effects indeed represent effects related to the dengue infection.

The children included in our study were all clinically severely ill; only these patients were included because gene array profiling studies investigating intra-individual gene transcription changes, such as differential activation of differential immunological pathways, are best performed in extreme cases. Not all children in our study could be identified as severe cases by WHO criteria: 7 patients (13%) were diagnosed as having DF. The limitations of the WHO criteria to identify all patients suffering from clinically severe disease have been observed before [49],[50]; to circumvent this problem, clinical associations were calculated not only for fulfilment of DSS criteria, but also for other disease severity parameters such as occurrence of complications, pleural effusion and hemorrhagic manifestations. No associations were calculated for mortality because of the low incidence (n = 4; 7%).

With the limitations of an observational, gene array profiling study in mind, the data from our study provide a first insight into the molecular basis of inflammatory gene expression patterns in peripheral blood leukocytes from children with severe dengue infections *in vivo*. The profile showed up-regulation of the antiviral cytokines *IFNG* and *IL12A* and down-regulation of the NF-κB pathway. The function of these differential gene expression patterns is not precisely clear, but may be related to multiple associations between the NF-κB pathway and adverse clinical outcomes. In addition to these data, it was found that the expression of several TLRs was affected during severe dengue disease.
